# Most Anti-BrdU Antibodies React with 2′-Deoxy-5-Ethynyluridine — The Method for the Effective Suppression of This Cross-Reactivity

**DOI:** 10.1371/journal.pone.0051679

**Published:** 2012-12-18

**Authors:** Radek Liboska, Anna Ligasová, Dmytro Strunin, Ivan Rosenberg, Karel Koberna

**Affiliations:** 1 Oligonucleotide Group, Institute of Organic Chemistry and Biochemistry, ASCR, v.v.i., Prague, Czech Republic; 2 Department of Molecular Cytology and Cytometry, Institute of Biophysics, ASCR, v.v.i., Brno, Czech Republic; 3 Department of RNA Biology, Institute of Molecular Genetics, ASCR, v.v.i., Prague, Czech Republic; Institute of Enzymology of the Hungarian Academy of Science, Hungary

## Abstract

5-Bromo-2′-deoxyuridine (BrdU) and 2′-deoxy-5-ethynyluridine (EdU) are widely used as markers of replicated DNA. While BrdU is detected using antibodies, the click reaction typically with fluorescent azido-dyes is used for EdU localisation. We have performed an analysis of ten samples of antibodies against BrdU with respect to their reactivity with EdU. Except for one sample all the others evinced reactivity with EdU. A high level of EdU persists in nuclear DNA even after the reaction of EdU with fluorescent azido-dyes if the common concentration of dye is used. Although a ten-time increase of azido-dye concentration resulted in a decrease of the signal provided by anti-BrdU antibodies, it also resulted in a substantial increase of the non-specific signal. We have shown that this unwanted reactivity is effectively suppressed by non-fluorescent azido molecules. In this respect, we have tested two protocols of the simultaneous localisation of incorporated BrdU and EdU. They differ in the mechanism of the revelation of incorporated BrdU for the reaction with antibodies. The first one was based on the use of hydrochloric acid, the second one on the incubation of samples with copper(I) ions. The use of hydrochloric acid resulted in a significant increase of the non-specific signal. In the case of the second method, no such effect was observed.

## Introduction

The detection of cellular DNA synthetic activity is a common approach used in a wide range of studies. It is commonly performed by labelling of DNA using 5-bromo-2′-deoxyuridine (BrdU; [1], [2]). BrdU is efficiently phosphorylated by cellular kinases and then incorporated in DNA strands by means of DNA polymerases. It is subsequently detected with anti-BrdU antibodies. Alternatively, 5-chloro-2′-deoxyuridine (CldU) or 2′-deoxy-5-iodouridine (IdU) can be used [3]–[5]. Because of the high similarity of CldU and IdU with BrdU, the anti-BrdU antibodies also react with these modified nucleosides [3]–[5]. Although it makes it possible to use them for the substitution of BrdU, it complicates the multiple labelling of cells.

Since the 5-halo-nucleosides incorporated in DNA are masked in the structure of double-stranded DNA, it is necessary to use special steps for their revelation [1], [2], [6]–[8]. These steps are based either on the partial degradation of DNA and/or DNA denaturation. The most commonly used approach depends upon depurination and the cleavage of DNA by strong mineral acids such as hydrochloric acid (HCl; [1], [2], [7]). The alternative approaches are based on the use of sodium hydroxide leading to the loosening of DNA as a consequence of the deprotonation of nucleobases [2], [6], [8] or on DNA cleavage by means of nucleases [1], [2]. A further alternative is the method based on the creation of gaps in DNA strands by the incubation of samples with monovalent copper ions in the presence of oxygen [9]. With respect to the signal strength, the most efficient methods are those based on strong acids and copper ions [9].

The alternative approach for the detection of DNA synthetic activity is based on the use of 2′-deoxy-5-ethynyluridine (EdU; [10]). Similarly to BrdU, EdU is effectively phosphorylated and subsequently incorporated in the newly-synthesised DNA strand. Its detection is based on the reaction of the terminal ethynyl with the azido group of the marker [10]. Although basically many molecules can serve as a marker, most commonly fluorescent azido-dyes are used.

In this study, we have analysed the possibility of the simultaneous employment of EdU and BrdU for the detection of DNA synthetic activity by means of various azido-dyes and antibodies. First, the affinity of ten different samples of anti-BrdU antibodies was tested using biotinylated molecules of EdU and BrdU bound to streptavidine-coated well plates. Subsequently, the antibodies were tested on fixed cells with EdU and/or BrdU incorporated. The obtained data showed the high affinity of the tested antibodies both to BrdU and EdU. This affinity persisted even after a click reaction with fluorochrome azido-dyes. We present here an approach enabling the effective suppression of the reactivity of antibodies with EdU. The method developed was tested for two protocols of concurrent revelation of the incorporated BrdU and EdU. The first protocol was based on the use of hydrochloric acid; the second one was based on the use of copper ions.

## Materials and Methods

### Preparation of the Biotinylated EdU, BrdU, and 2′-deoxythymidine and their Attachment to Streptavidine-coated Well Plates

The 5-substituted 2′-deoxy-5′-*O*-dimethoxytrityluridine-3′-*O*-yl hemisuccinates were attached to the solid support (Amino-SynBase™ CPG 500/110) and coupled with 1-dimethoxytrityloxy-3-*O*-(*N*-biotinyl-3-aminopropyl)-triethyleneglycolyl-glyceryl-2-*O*-(2-cyanoethyl)-(*N,N*-diisopropyl)-phosphoramidite (Glen Research) using the standard phosphoramidite protocol on an automated DNA/RNA synthesiser by the ‘trityl on’ method. The desired products were purified after removing them from the support with pressured gaseous ammonia (100 psi, 2 h, r.t.) and deblocking them in 75% aq. acetic acid (30 min at r.t.), on the reversed phase column (Luna C18, 5 µm, 10×250 mm, 3 ml/min, gradient 0→50% acetonitrile in 0.05 M-ammonium hydrogencarbonate) using an LCMS device (Autopurification System, Waters).

The well plates (with a binding capacity of ∼125 pmol of biotin per well) were washed three times with a Tris buffer (25 mM Tris-HCl, pH 7.2; 150 mM NaCl; 0.1% BSA; 0.05% Tween-20) and incubated with 1 nmol of the ligand per well (100 µl of 10 µM solution; 2 hrs. at room temperature). After incubation, the well plates were washed three times with Tris-buffer and immediately used for the antibody assay.

### Cell Culture, DNA Labelling, Fixation and Permeabilisation Protocol

Human HeLa cells (a generous gift from Dr. David Staněk, Institute of Molecular Genetics, Prague) were cultured on coverslips in a Petri dish in Dulbecco’s modified Eagle’s medium with L-glutamine (DMEM, Gibco) supplemented with 10% foetal calf serum (PAA Laboratories), 1% gentamicin and 0.85 g/l of NaHCO_3_ at 37°C in a humidified atmosphere containing 5% CO_2_.

For the labelling of DNA replication, BrdU or EdU were added to the culture medium for 10 minutes if not stated otherwise. The final concentration of BrdU and EdU was 20 µM.

The cells were fixed with 2% formaldehyde for 10 minutes, washed in 1× PBS, permeabilised in 0.2% Triton X-100 for 10 minutes and washed in 1× PBS.

### BrdU Revelation

The cells were incubated either with 4N HCl for 20 minutes at room temperature or in a freshly-prepared solution of 10 mM sodium ascorbate, and 4 mM copper(II) sulfate for 10 minutes and then in 20 mM EDTA for 30 minutes at room temperature. In the latter case, the primary antibodies were diluted in 1× buffer for exonuclease III (Fermentas) and supplemented by exonuclease III (0.1 U/µl; Fermentas). The revelation of BrdU by means of copper(I) ions is based on the introduction of frequent gaps into DNA by oxidative attack at the deoxyribose moiety and subsequent prolongation of the gaps by exonuclease activity. According to our findings, this method in contrast to the treatment of cells by hydrochloric acid does not result in the progressive release of DNA and DNA-bound proteins [9].

### Detection of EdU and BrdU

The cells or well plates were incubated with primary antibodies for 30 minutes at room temperature and washed in Tris buffer. When employing copper(I) ions for BrdU revelation, we used 1× PBS instead of the Tris buffer. The plates or cells were afterwards incubated with secondary antibodies for 30 minutes, washed in the Tris buffer or 1× PBS and washed in distilled water. The following monoclonal and polyclonal primary antibodies were used in the study: BMC 9318 (mouse, Roche), B44 (mouse, Becton Dickinson), Bu20a (mouse, BioLegend), BU-33 (mouse, Sigma Aldrich), BU6-4 (mouse, Genetex), BU5.1 (mouse, Millipore), MoBu-1 (mouse, Exbio) and BU1/75 (rat, Abcam), chicken polyclonal anti-BrdU antibody (Abcam) and sheep polyclonal anti-BrdU antibody (Genetex). The primary antibodies were diluted to the final concentration of 2.5 µg/ml in the Tris buffer or in 1× PBS. The Tris buffer contained 25 mM Tris-HCl, pH 7.2, 150 mM NaCl, 0.1% BSA and 0.05% Tween 20. The 1× PBS buffer contained 137 mM NaCl, 3 mM KCl, 16 mM Na_2_HPO_4_, 2 mM KH_2_PO_4_, pH 7.4, 0.1% BSA and 0.05% Tween 20. When employing copper(I) ions for BrdU revelation, BSA and Tween 20 were omitted.

The following secondary antibodies were used: Cy3 anti-mouse, Cy3 anti-rat, Cy3 anti-chicken, Cy3 anti-sheep, FITC anti-rat, and DyLight 649 anti-rat antibodies (Jackson Immunoresearch). The secondary antibodies were diluted 1∶100 in 1× PBS (copper(I)-treated cells) or the Tris buffer (HCl-treated cells and well plates).

### Azido-molecules, Click Reaction and EdU Blocking

The following azido-dye molecules were used: Alexa Fluor® 488 azide (0.02 mM or 0.2 mM), Alexa Fluor® 555 azide (0.2 mM), Alexa Fluor® 647 azide (0.2 mM; all Invitrogen), Cy5 azide (0.2 mM), TAMRA azide (0.2 mM), Cy3.5 azide (0.2 mM), 5-FAM azide (0.2 mM), and 6-ROX azide (0.2 mM; all Lumiprobe). For EdU blocking, we used 2-azidoethanol (0.02 mM, 0.2 mM, 2 mM and 20 mM) synthesised from bromoethanol and sodium azide in water according to the described procedure [11], racemic 1-azido-2,3-dihydroxypropane (0.02 mM, 0.2 mM, 2 mM and 20 mM) prepared from glycidol, sodium azide and ammonium chloride according to the procedure of [12] and azidomethylphenylsulfide (azidosulfide; 0.02 mM, 0.2 mM, 2 mM; Sigma Aldrich). The click reaction was performed using the commercially available kit (Invitrogen) for 30 minutes at room temperature. The blocking of EdU was performed with the kit from Invitrogen.

### Well-plate Analysis, Image Acquisition and Processing

Reacti-Bind™ Streptavidin Coated High Binding Capacity (HBC) Black 96-Well Plates (Thermo Scientific Pierce Prod # 15503) were used for the fluorescence measurements on an Infinite® F500 multimode microplate reader (Tecan).

The images from fluorescence microscopy were obtained using an Olympus IX81 microscope (Olympus) equipped with a Hamamatsu ORCA II camera with a resolution of 1344×1024 pixels using Cell∧R acquisition software. The UPLFLN oil 40× NA 1.3 objective was used. For the image processing, Adobe Photoshop software was used.

For the evaluation of the fluorescence signal intensities, two methods were used. The first method was previously used in [13] and is based on the analysis of the minimal times necessary for the appearance of the first signs of saturation in any region of the image. The signal intensity is therefore inversely proportional to the acquisition times. Twenty images were evaluated for every sample using this method. The second method is based on the evaluation of the mean intensities of the signal from cell nuclei acquired for the same time. Twenty images were acquired from every sample and twenty nuclei were evaluated in every such image (400 nuclei in total).

## Results

### Most of the Primary Antibodies Against BrdU React also with EdU

**Figure 1:**
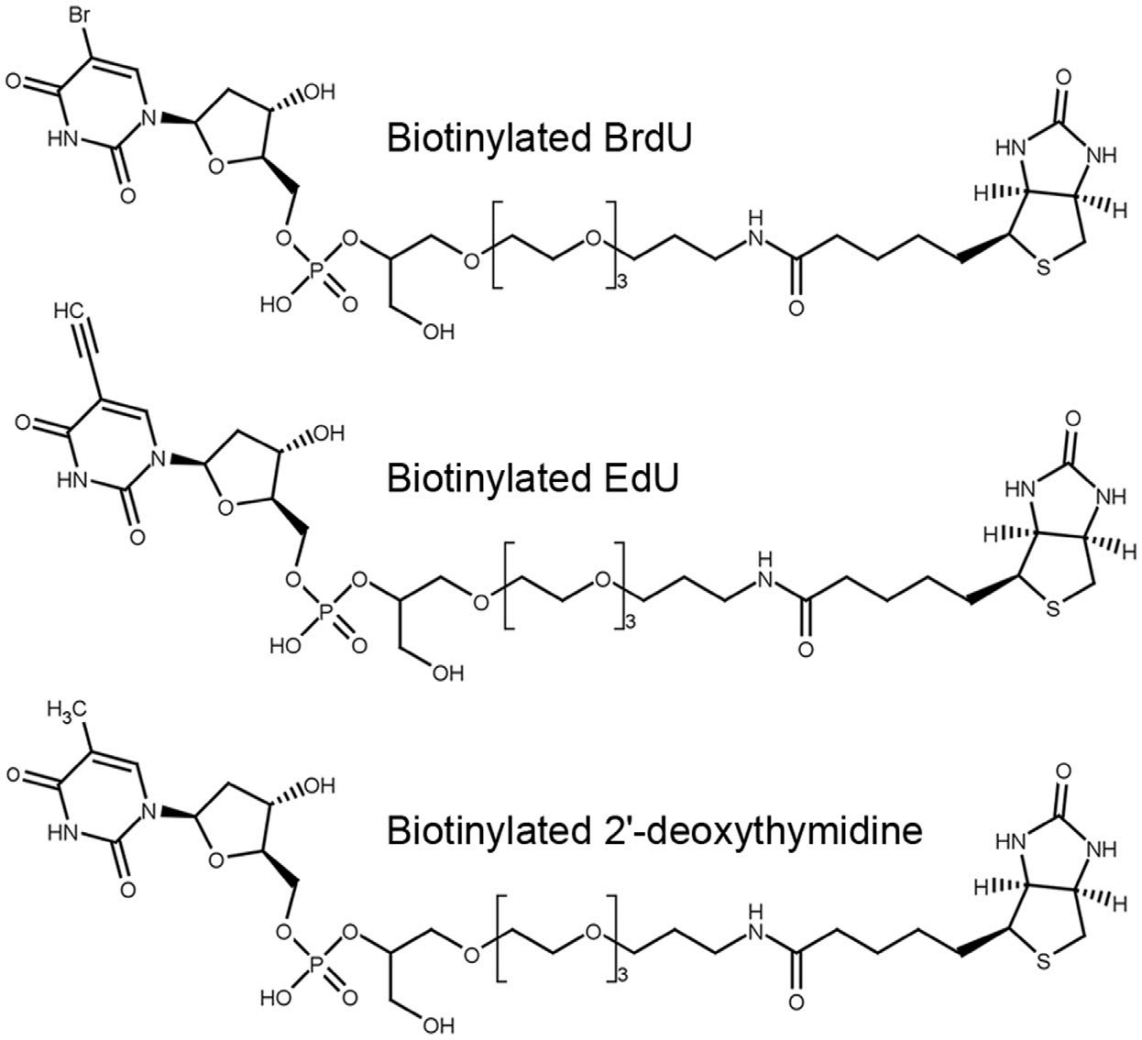
The structure of biotinylated nucleoside ligands. The picture shows the chemical structure of biotinylated BrdU, EdU and 2′-deoxythymidine ligands.

**Figure 2:**
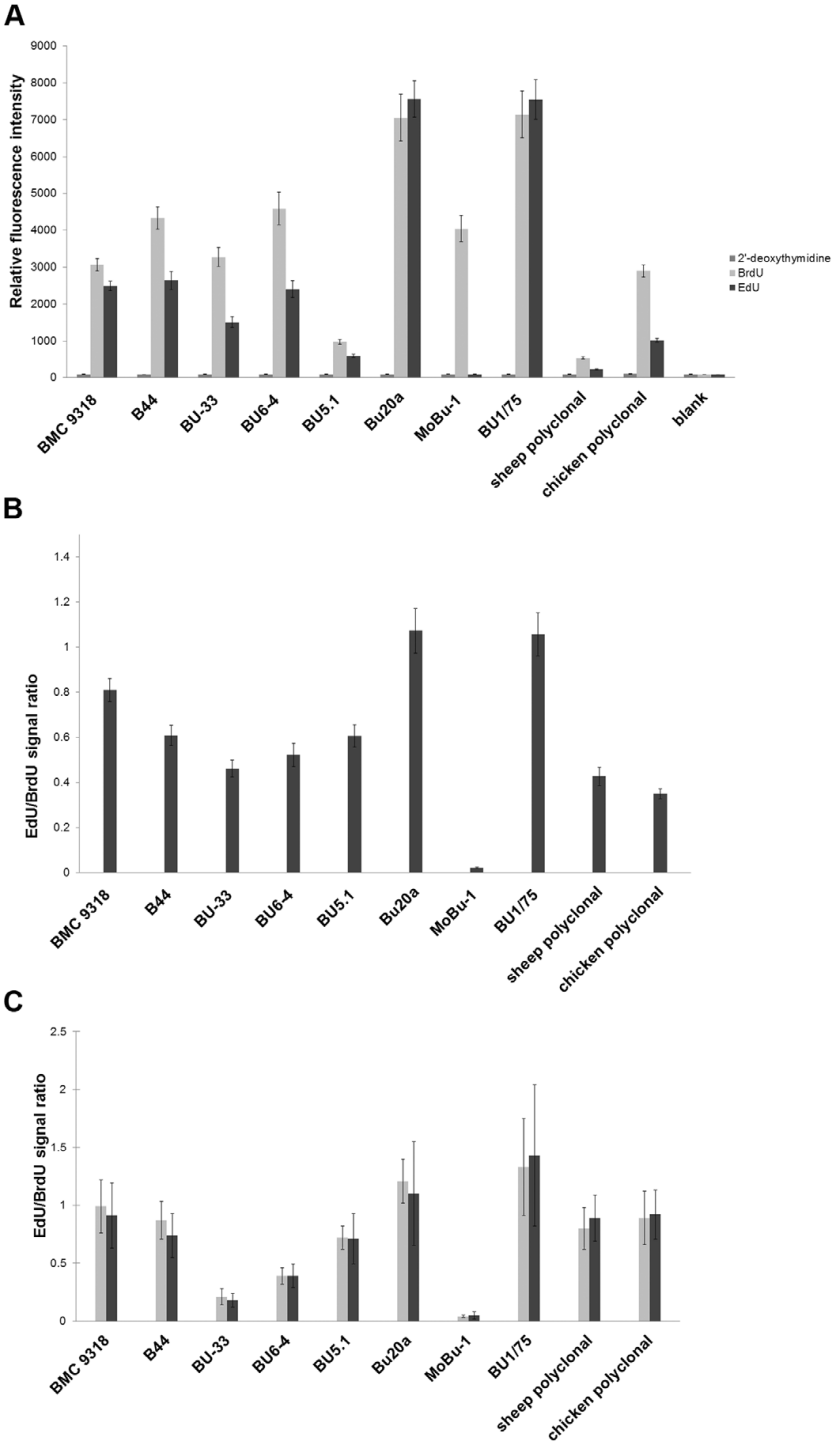
The affinity of antibodies to BrdU, EdU and 2′-deoxythymidine on well plates and in cellular DNA. A) A comparison of the relative fluorescence intensity after the reaction of ten primary antibodies with BrdU, EdU and 2′-deoxythymidine anchored to the streptavidin-coated well plates. B) The ratio between the EdU and BrdU signals for streptavidin-coated well plates. C) The ratio between the EdU and BrdU signals for nuclear DNA after a two-hour incorporation of BrdU or EdU. The signal strength was determined according to ([13]; light grey columns) or by the evaluation of the mean intensities of the signal of labelled nuclei (dark grey columns, see also Material and Methods). Besides the antibody clone MoBu-1, all the other antibodies reacted both with BrdU and EdU.

First, the prepared biotinylated BrdU, EdU and 2′-deoxythymidine were incubated with a streptavidin-coated 96-well plate and afterwards with anti-BrdU antibodies. The bound anti-BrdU antibodies were detected by the appropriate secondary antibodies. The structures of the biotinylated nucleosides are depicted in Fig. 1. In order to preserve the maximum accessibility of a nucleoside for the reaction with antibody molecules, we used a long triethyleneglycolylglycerolylphosphate linker. The results of this analysis are summarised in the graphs (Fig. 2A and 2B). It is evident that with the exception of the antibody clone MoBu-1, the rest of the tested antibodies reacted both with BrdU and EdU.

**Figure 3:**
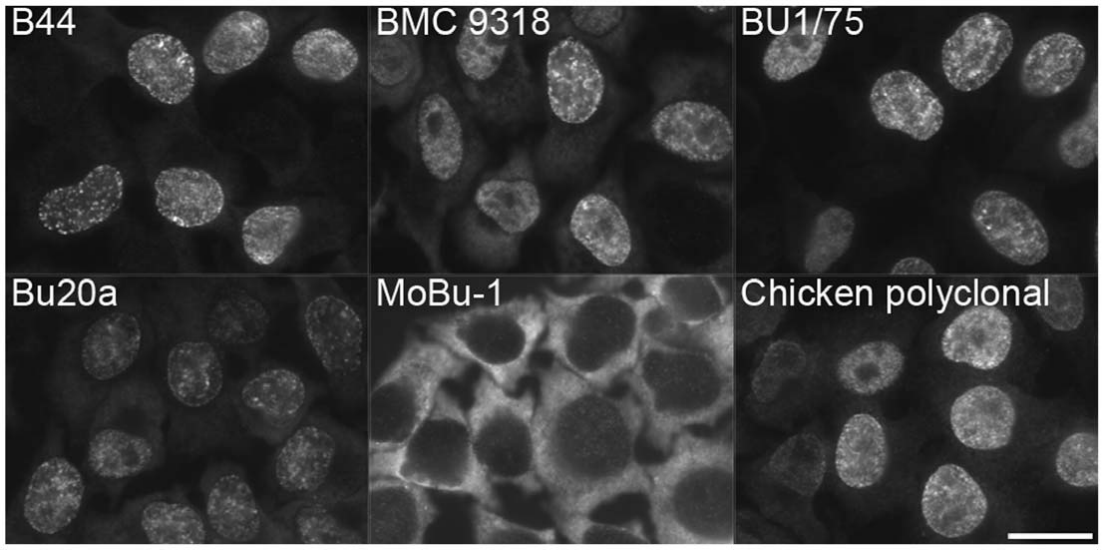
The detection of EdU in fixed HeLa cells. The picture shows examples of the detection of EdU in fixed and permeabilised cells with six antibodies after a ten-minute EdU labelling pulse. Note that only the clone MoBu-1 exhibits no signal. Barr: 20 µm.

Second, we tested the anti-BrdU antibodies on the fixed and permeabilised cells after pulses of EdU or BrdU (Fig. 2C and 3). For EdU and BrdU revelation, we used the incubation of cells in 4N HCl. The performed experiments confirmed the results obtained on the well plates. The only exception was the antibody clone BU-33. Under the conditions used, this antibody reacted neither with BrdU nor with EdU. On the other hand, this clone reacted both with BrdU and EdU after the prolongation of the BrdU or EdU incorporation time. 2-hour incorporation was sufficient to detect BrdU and EdU in nuclear DNA. Therefore, we tested the relative reactivity of all the antibody clones with BrdU and EdU in the HeLa cell nuclei after a 2-hour incorporation of BrdU or EdU (Fig. 2C). We used two methods to evaluate the reactivity of particular antibodies with BrdU and EdU in nuclear DNA (see Material and Methods). Using the first one, we determined the EdU/BrdU signal ratio as the ratio between the time length necessary to achieve the first signs of saturation in the image of the BrdU-labelled cells and the image of the EdU-labelled cells. Alternatively, we used the ratio between the mean intensity of the images of the nuclei of EdU-labelled cells and BrdU-labelled cells (see Material and Methods). With the exception of the clones BU1/75 and Bu20a, the signal intensity was stronger for BrdU than for EdU both in the case of well plates and fixed cells. We also tested the omission of BSA and Tween 20 in the Tris-HCl buffer. It resulted in a change of the EdU/BrdU signal ratio. We found that the highest changes of the EdU/BrdU signal ratio deduced from the mean signal of the labelled nuclei were observed in the case of clone BU6-4 (0.39±0.1 or 1.01±0.52 with or without BSA and Tween 20, respectively) and BU1/75 (1.43±0.61 or 0.67±0.25 with or without BSA and Tween 20, respectively). These data indicated that there was no clear dependence between the EdU/BrdU signal ratio and the presence of Tween 20 and BSA in the buffer. On the other hand, we did not observe the influence of the exchange of a Tris-HCl buffer for a PBS buffer (data not shown).

### A Significant Portion of EdU Still Remains in the Cellular DNA after the Reaction with Fluorochrome Azido-dyes

In the next experiments, we performed immunodetection of EdU in cells after the click reaction with Alexa Fluor® 488 azide and incubation with 4N HCl. The detections were performed with the antibody clone BU1/75. This clone together with the clone Bu20a evinced the strongest signal intensity in the case of the EdU detection at well plates (see Figure 2) and provided the highest signal/noise ratio in the case of both BrdU and EdU detection in the permeabilised cells. Surprisingly, the click reaction did not significantly decrease the signal. Moreover, the incubation of cells in an acid environment resulted in a non-specific signal in the nucleoli derived from fluorescent azido-dyes. Similar data were obtained when the acid treatment preceded the click reaction. We observed no significant effect on the antibody-derived signal and the non-specific labelling of nucleoli. However, this protocol led also to an observable decrease of the signal provided by Alexa Fluor® 488 azide. Similar results were also obtained when we used the antibody clone BMC 9318.

**Figure 4:**
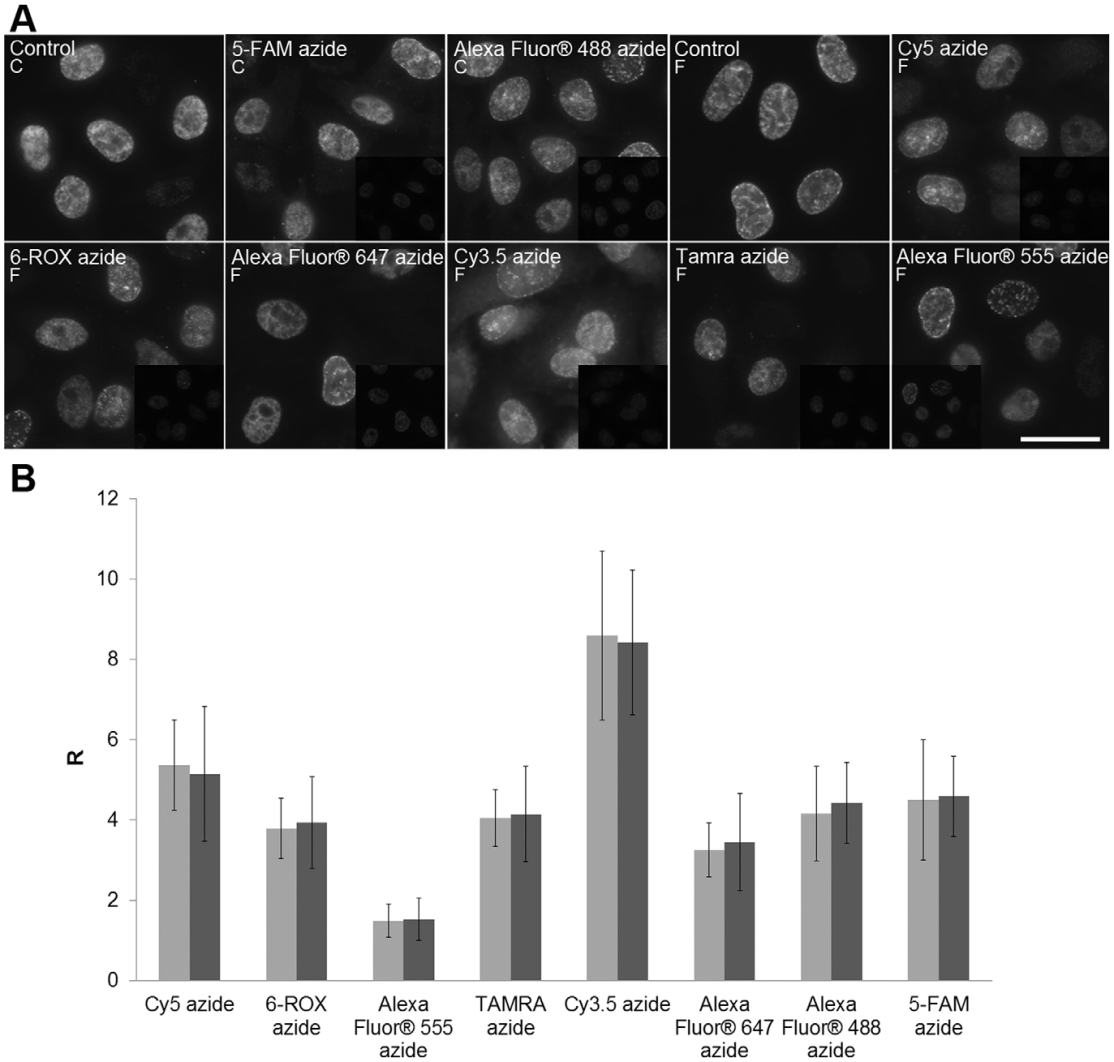
The detection of EdU after the click reaction with various fluorescent azido dyes. A. The results of the detection of EdU using the antibody clone BU1/75 and anti-rat antibody conjugated with FITC (F) or Cy3 (C) after the click reaction with 0.2 mM fluorescent azido-dyes or without the click reaction (control) in fixed and permeabilised cells labelled for 10 minutes with EdU are shown. The cells were incubated with 4N HCl prior to the click reaction. The images were acquired for various times in order to demonstrate that the EdU-derived signal of the anti-BrdU antibody is not completely removed by the click reaction with an elevated concentration of azido-dyes. The images in the inserts were acquired for the same time (190 ms for cells labelled with anti-rat antibody FITC conjugate and 74 ms for cells labelled with anti-rat Cy3 conjugate). Their intensity therefore reflects the decrease of the EdU-derived signal of the anti-BrdU antibody signal after the click reaction with respect to the control cells. Barr: 20 µm. **B.** The level of the suppression of the signal is shown for the individual azido dyes as the ratio (R) between the time length necessary to achieve the first signs of saturation in the image of the evaluated sample and in the image of control sample without the click reaction (light grey columns). Alternatively, we used the ratio between the mean intensity of the images of nuclei of the control cells and sample cells (dark grey columns).

Further, we analysed the EdU-derived signal of clone BU1/75 after the click reaction with a ten times higher concentration of fluorescent azido-dyes (0.2 mM). We tested eight different fluorescent azido-dyes (Fig. 4). In the control experiments, the cells were labelled with EdU for 10 minutes, fixed, permeabilised, the DNA was denatured with 4N HCl and the cells were incubated with an anti-BrdU antibody (clone BU1/75) and an anti-rat antibody conjugated with FITC or Cy3. In the sample cells, the click reaction with fluorescent azido-dyes was performed after the denaturation step (4N HCl) and the cells were incubated with an anti-BrdU antibody (clone BU1/75) and then with an anti-rat antibody conjugated with FITC or Cy3.

**Figure 5:**
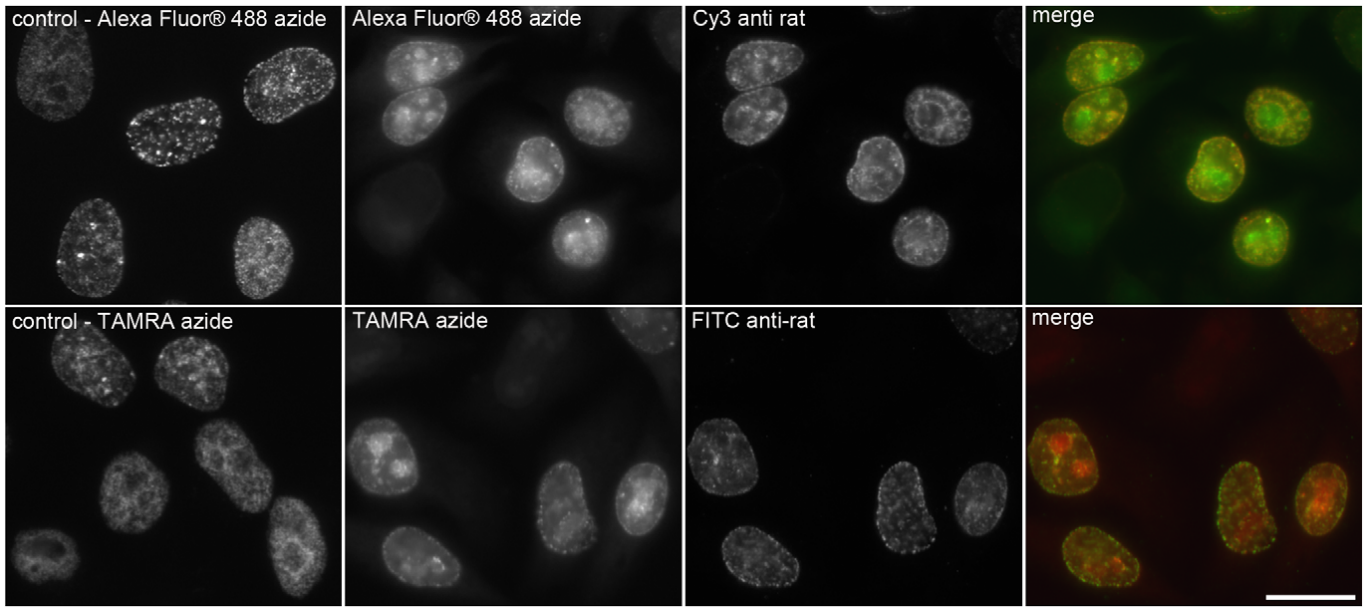
The increase of azido dye concentration and acid treatment results in a non-specific signal. The picture shows examples of the detection of incorporated EdU in DNA by means of a click reaction with 0.02 mM fluorescent dye without the HCl treatment (images labelled as control) and of detection with 0.2 mM fluorescent azido dyes and the antibody clone BU1/75 (Cy3 anti-rat and FITC anti-rat secondary antibodies) in cells treated with 4N HCl. Note that the higher concentration of fluorescent azido dyes and the subsequent treatment with 4N HCl led to a non-specific signal mainly in the nucleolus area. Barr: 20 µm.

Although the increase of the concentration of dyes led to a significant decrease of the signal in all cases, it never resulted in its complete suppression (Figure 4). The decrease was different for every dye. To evaluate the rate of the signal reduction, we used the ratio between the time length necessary to achieve the first signs of saturation in the image of the sample and the image of the control cells. Alternatively, we used the ratio between the mean intensity of images of the nuclei of the control cells and sample cells (Fig. 4B). The increase of the dye concentration generally resulted also in a substantial increase of the non-specific fluorescence. It was especially apparent in the area of the nucleoli (Fig. 5). This fluorescence signal was not possible to remove even after a one-hour washing of the cells in a Tris-HCl buffer or PBS buffer.

**Figure 6:**
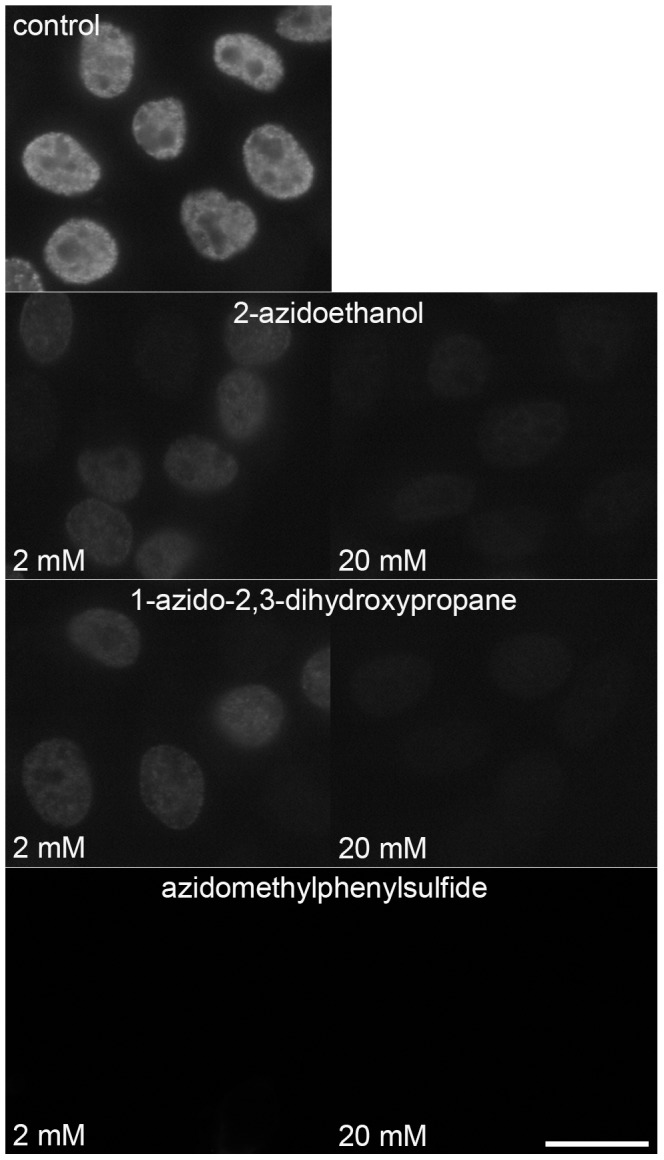
The suppression of the EdU signal using non-fluorescent azido molecules. The suppression of the signal provided by BU1/75 antibody after a click reaction with 2 or 20 mM 2-azidoethanol, 1-azido-2,3-dihydroxypropane or azidomethylphenylsulfide in cells labelled with EdU for 20 minutes. The images were acquired for 8 ms. Barr: 20 µm.

As it was evident that the fluorescent dyes could not be used for the suppression of the EdU-derived signal, we used three non-fluorescent azido-molecules: 2-azidoethanol, 1-azido-2,3-dihydroxypropane and azidomethylphenylsulfide. We tested their effect on the suppression of the signal using the BU1/75 antibody (Fig. 6). The obtained data showed that while the 2 mM azidomethylphenylsulfide is enough for complete suppression of the signal, the same concentration of 2-azidoethanol and 1-azido-2,3-dihydroxypropane led only to a substantial decrease of the signal (Fig. 6). Complete suppression of the signal was observed after the application of 20 mM 2-azidoethanol or 1-azido-2,3-dihydroxypropane.

**Figure 7:**
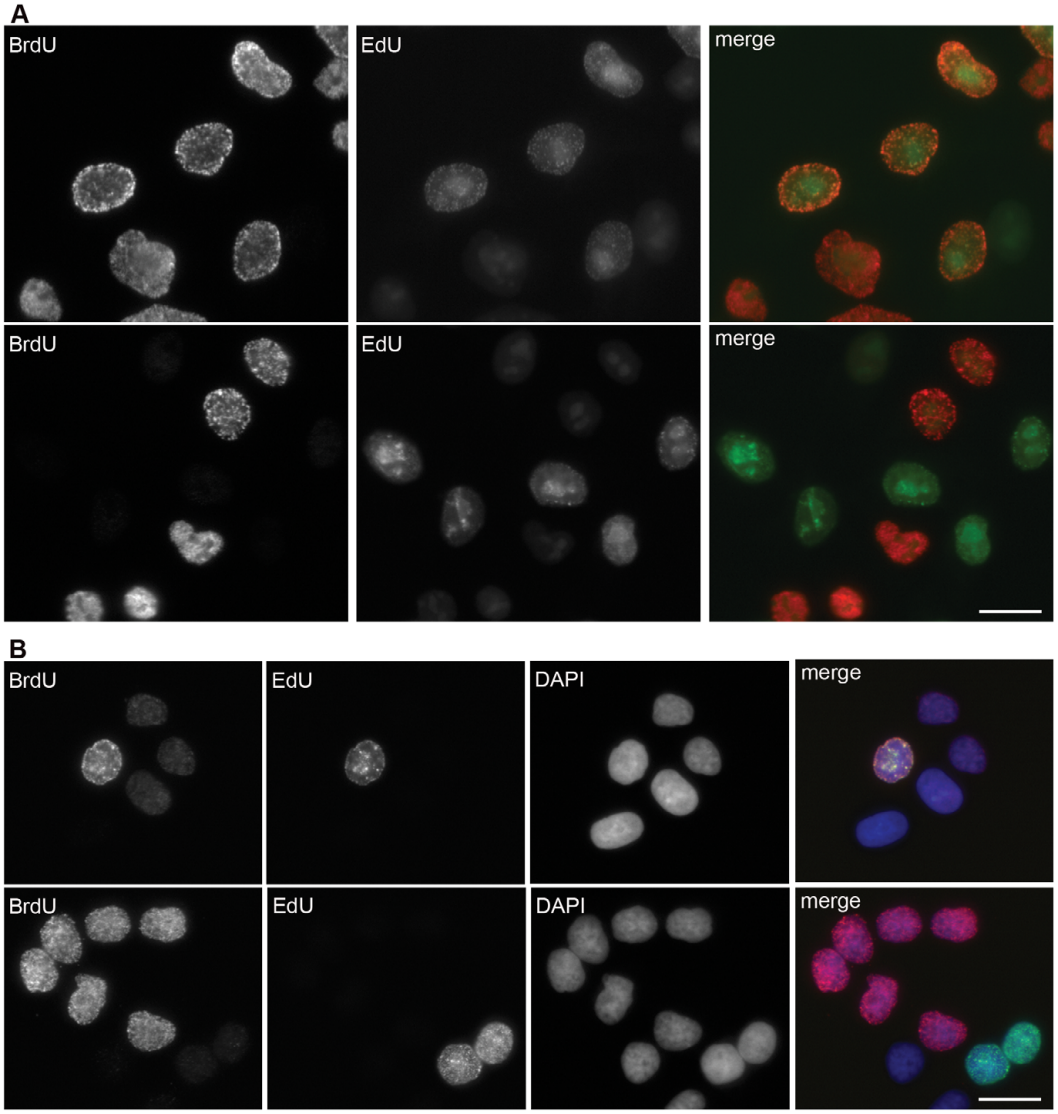
The simultaneous localisation of BrdU and EdU. The results of simultaneous localisation of BrdU and EdU using two different protocols for BrdU revelation in cells labelled for 5 minutes with BrdU and then after 13 hours for 20 minutes with EdU. **A.** The revelation of BrdU by 4N HCl is shown in the picture. The upper set of images represents the detection of both signals without the application of the blocking step by means of 2 mM azidomethylphenylsulfide. The bottom set of images represents the detection of both signals with the application of the blocking step by means of 2 mM azidomethylphenylsulfide. Barr: 20 µm. **B.** The revelation of BrdU by copper(I) ions and exonuclease III is shown in the picture. The upper set of images represents the detection of both signals without the application of the blocking step by means of 2 mM azidomethylphenylsulfide. The bottom set of images represents the detection of both signals with the application of the blocking step by means of 2 mM azidomethylphenylsulfide. Barr: 20 µm.

Further, we tested the use of azidomethylphenylsulfide for the simultaneous labelling of the cells labelled with EdU and BrdU. We incubated the cells in a culture medium supplemented with 20 µM BrdU for five minutes and then in a medium supplemented with 20 µM 2′-deoxythymidine for one hour. The addition of 2′-deoxythymidine was used to minimise the time of BrdU incorporation after the five-minute BrdU pulse. After 2′-deoxythymidine removal, the cells were incubated for twelve hours in a fresh medium. Then, 20 µM EdU was added to the culture medium for 20 minutes. Considering the eight- to ten-hour length of the S phase of the used HeLa cell line and the 24-hour length of the cell cycle, the majority of the cells contained either BrdU or EdU in their DNA. The detection of EdU was performed with Alexa Fluor® 488 azide (Fig. 7). For BrdU revelation, we used either a protocol based on 4N HCl (Fig. 7A) or on copper(I) ions and exonuclease III (Fig. 7B). The incorporated BrdU was detected by means of the BU1/75 antibody (Fig. 7). From the results obtained, it is obvious that the click reaction with 2 mM azidomethylphenylsulfide led to a complete de-localisation of both signals. In contrast, the protocol without the blocking step resulted in an overlap of the BrdU- and EdU-derived signals. In this respect, both protocols for BrdU revelation provided the same results. On the other hand, the acid treatment resulted in a non-specific fluorescence of Alexa Fluor® 488 especially in the nucleoli area and a decrease of the azido-dye signal. No non-specific signal and only a subtle if any decrease of the azido-dye signal was observed in the cells incubated in the solution of copper(I) ions.

## Discussion

Ten antibodies directed against BrdU were tested in this study. Besides eight monoclonal antibodies, we also tested two polyclonal antibodies. Only one of the tested antibodies did not react with EdU (clone MoBu-1). This finding is in accordance with the finding of [14], who also showed that this antibody clone does not react with EdU. The rest of the tested antibodies showed strong reactivity with EdU, which was preserved even after a click reaction of EdU with an azido dye in fixed HeLa cells. Although EdU reacted with the tested antibodies more weakly than BrdU in almost all cases, the affinity was still very high. Surprisingly, two antibody clones had higher affinity to EdU than to BrdU. Although in the case of the detections of EdU in cellular DNA the possibility that EdU is incorporated in DNA with a different efficiency than BrdU e.g. due to the different efficiency of its phosphorylation or the earlier published inhibition effect on the thymidylate synthase [15] has to be taken into consideration, this effect is not applicable for the data obtained from well plates. Most importantly, the complete suppression of the EdU signal derived from anti-BrdU antibodies was not observed even when the concentration of azido dyes was increased by ten fold during the click reaction step. It was also evident that the particular azido dyes suppressed this signal with different efficiency. The use of such high concentrations of azido dyes led to a relatively high non-specific signal, mainly in the area of the nucleoli. That is why we tested three non-fluorescent azido molecules: azidoethanol, 1-azido-2,3-dihydroxypropane and azidomethylphenylsulfide. Surprisingly, although azidomethylphenylsulfide is much larger than the simultaneously tested 2-azidoethanol and larger than 1-azido-2,3-dihydroxypropane, it turned out to be much more effective than 2-azidoethanol or 1-azido-2,3-dihydroxypropane. In this respect, it is evident that generally other azido molecules can also serve as effective blocking agents and their efficiency is not purely proportional to the size of these molecules.

We tested two basic protocols for the concurrent detection of BrdU and EdU by means of azidomethylphenylsulfide. The effective suppression of the EdU signal was observed in the case of both protocols. The only, yet important, differences were the presence of the non-specific fluorescence in the nucleoli area and the general significant decrease of the azido-dye signal in the case of the application of hydrochloric acid. From this point of view, it is more favourable to use the protocol based on the cleavage of DNA by monovalent copper ions followed by exonuclease cleavage. Although presently it is possible to use a 2′-deoxy-5-ethynylcytidine [16] as a second marker for the visualisation of DNA replication, it is not clear whether this modified nucleoside may be partially converted into EdU by cellular enzymes and then incorporated in DNA in this form as well.

Presently, only one anti-BrdU antibody that does not react with EdU is commercially available (mouse clone MoBu-1). Therefore, the blocking of EdU by non-fluorescent azido-molecules can be used especially in those cases when the simultaneous labelling of DNA by means of EdU and BrdU and the immunodetection of an additional cellular component by mouse antibodies is required. Importantly, the mouse is a very common source of monoclonal antibodies, including antibodies against the proteins playing a role in the DNA replication. In this respect, the frequently used rat clone BU1/75 exhibited very strong reactivity with EdU. Basically, the blocking step should also enable the triple labelling of DNA by EdU, CldU and IdU; as for CldU and IdU labelling, the antibodies that strongly react with EdU could be used (clone BU1/75 and clone B44, for the protocol see e.g. [3]). In this respect, our results strongly indicate that the blocking of EdU by non-fluorescent azido molecules enables the use of any clone of anti-BrdU antibodies without the need to test its reactivity with EdU.

Taking all the results together, it is clear that the most useful protocol for the blocking of EdU binding is based on the use of non-fluorescent azido-molecules. Their use is not accompanied by a non-specific signal and therefore extensive washing is not necessary. In addition, the commercially available variants are much cheaper than fluorescently-labelled azido dyes. The choice of a non-fluorescent molecule can also depend on the solubility of the azido-molecule. In this respect, the azidomethylphenylsulfide is soluble in DMSO, but only a low concentration can be obtained by its dissolution in water. In contrast, 2-azidoethanol or 1-azido-2,3-dihydroxypropane are quickly dissolved in water and a 100 mM stock solution can be prepared easily. The second important prerequisite for the best result is the use of the protocol that minimises the destruction of cell structure and especially DNA. According to our results, the use of hydrochloric acid results in a decrease of the EdU signal after the click reaction with an azido dye and in a non-specific signal especially in the nucleolar area. This protocol also does not enable the simultaneous use of such DNA dyes as DAPI or Hoechst. In this respect, the protocol based on copper ions is the superior method.

Generally, the following protocol provides good results of EdU blocking and BrdU revelation : The fixation of the cells with 2% formaldehyde (10 minutes), brief washing (PBS; 3 times), permeabilisation of the cells with 0,2% Triton X-100 (10 minutes), brief washing (PBS, 3 times), the click reaction with a fluorescent azido dye for 30 minutes, brief washing (PBS, 3 times), the click reaction with a non-fluorescent molecule for 30 minutes, incubation of the cells in a freshly-prepared solution of 10 mM sodium ascorbate, and 4 mM copper(II) sulfate for 10 minutes and then in 20 mM EDTA for 30 minutes. After a brief washing in PBS (3 times), BrdU can be detected by means of anti-BrdU antibodies in the presence of exonuclease III and DNA can be stained by means of DNA-specific dyes (e.g. DAPI).
